# Tethered and Polymer Supported Bilayer Lipid Membranes: Structure and Function

**DOI:** 10.3390/membranes6020030

**Published:** 2016-05-30

**Authors:** Jakob Andersson, Ingo Köper

**Affiliations:** Flinders Centre for Nanoscale Science and Technology, School of Chemical and Physical Sciences, Flinders University, Adelaide SA 5001, Australia; jakob.andersson@flinders.edu.au

**Keywords:** solid supported membrane, model membrane, membrane structure and function

## Abstract

Solid supported bilayer lipid membranes are model systems to mimic natural cell membranes in order to understand structural and functional properties of such systems. The use of a model system allows for the use of a wide variety of analytical tools including atomic force microscopy, impedance spectroscopy, neutron reflectometry, and surface plasmon resonance spectroscopy. Among the large number of different types of model membranes polymer-supported and tethered lipid bilayers have been shown to be versatile and useful systems. Both systems consist of a lipid bilayer, which is de-coupled from an underlying support by a spacer cushion. Both systems will be reviewed, with an emphasis on the effect that the spacer moiety has on the bilayer properties.

## 1. Introduction

Biological membranes and membrane related processes are essential for every living organism. They form the outer layer of cells and organelles and host a large variety of proteins that are essential to the functioning of every cell. G-protein coupled receptors, for example, are involved in a wide range of cellular processes such as vision [1], smell [2], and cardiac function [3,4]. Malfunction of membrane proteins can cause severe diseases such as cystic fibrosis [5] or muscular dystrophy [6]. Currently, most novel pharmaceuticals target G-protein coupled receptors [7], which makes this class of membrane proteins the most important drug target ever discovered [8]. Furthermore, the cell membrane serves as the outermost barrier of the cell against pathogens. To enter a cell and cause damage, pathogens and toxins will have to interact with or pass through the cell membrane [9].

**Figure 1:**
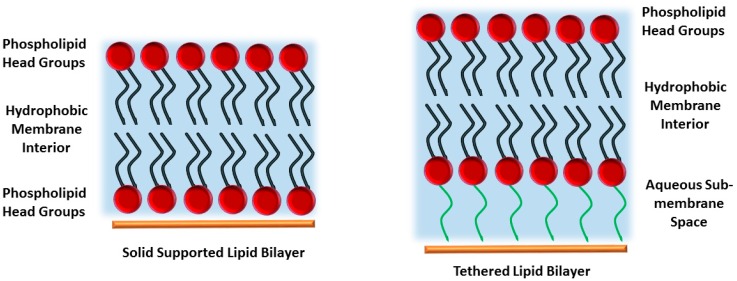
Solid supported bilayer (**left**) and tethered lipid bilayer membrane (tBLM, **right**). While a solid supported membrane consists of a lipid bilayer floating on a solid support, in a tBLM the proximal leaflet is covalently grafted to the substrate, providing a sub-membrane space as well as a more stable architecture.

Systematic studies of membrane processes and the detailed structure of lipid membranes are hindered by the intrinsic complexity of their architecture, which comprises a wide variety of lipids, proteins, and carbohydrates. A large number of different model systems have been developed that reduce the natural complexity of the membrane, while providing an easily accessible experimental platform. These systems mimic essential structural and chemical aspects of a lipid bilayer membrane and include Langmuir monolayers, vesicles, bilayer (or black) lipid membranes, and solid supported bilayer lipid membranes (sBLMs) (Figure 1) [10,11,12,13,14].

Among the different model systems, sBLMs and tethered bilayer lipid membranes (tBLMs) have attracted significant interest because they offer a relatively stable platform and are accessible to a wide range of analytical techniques, which gives the potential for them to be used in a wide variety of applications.

Solid supported bilayer lipid membranes consist of a lipid bilayer that is placed on a solid support [10]. In their most simple form, the membrane is in direct contact with the surface, typically separated from the support only by a thin layer of water with a thickness of less than a few Ångström [14,15,16]. Compared to free standing bilayers, sBLMs allow for the use of a wider range of analytical techniques, including surface sensitive techniques such as surface plasmon resonance or atomic force microscopy to study membrane properties. The solid support also provides increased stability compared to other model systems. sBLMs have been used to study various membrane related processes such as the influence of lipid composition on the bilayer structure [17,18,19], the binding of peptides and proteins to the bilayer [20], and the interaction of phospholipid bilayers with small molecules such as sterols [21] and peptides [22]. Solid supported membranes can also be used to investigate the binding of drugs or drug-like molecules to the membrane or to receptors within the membrane. However, a bilayer floating on a solid support is still relative unstable and makes long-time measurements requiring days or weeks very difficult. The small amount of available space underneath the membrane also hinders protein incorporation, as incorporated proteins are likely to interact with the support [11]. This has been found to cause proteins to denature, making structural or functional studies impossible.

As an extension of sBLMs, polymer supported and tethered lipid bilayer membranes have been developed. These model systems separate the lipid bilayer from the substrate by a spacer moiety. Here, polymer supported and tethered bilayer lipid membranes will be reviewed with a specific focus on the influence of the sub-membrane architecture on membrane properties.

## 2. Polymer-Supported Lipid Bilayers

**Figure 2:**
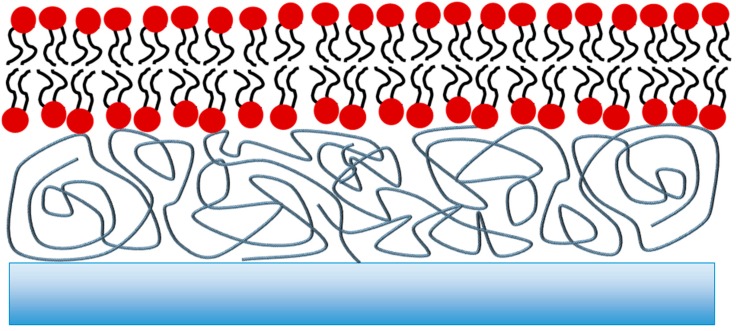
Schematic of a polymer-tethered bilayer. The lipid bilayer is deposited on a cushion comprised of a polymer usually covalently attached to the support. While a well-hydrated sub-membrane reservoir can be achieved, the bulk and chemical nature of the polymer chains may affect protein incorporation and transport studies.

To reduce membrane-substrate interactions, the bilayer can be separated from the support by the introduction of a polymer layer (Figure 2) [11,23]. This layer is typically bound to the surface of the support material first and the lipid bilayer is subsequently deposited onto the polymer cushion. The cushion, if appropriately designed, can also mimic the cytoskeleton of a cell. However, the sub-membrane reservoir thickness depends on the polydispersity of the polymer used as well as its swelling behaviour upon hydration. Both factors are difficult to control with absolute precision and therefore the resulting bilayers have been shown to often contain holes and defects, making polymer-supported bilayers generally less favourable to be used as biosensing platforms [24,25].

Furthermore, the nature and density of the polymer support has been shown to significantly affect membrane properties. For example, the diffusion of embedded lipids and proteins is significantly affected, resulting in anomalous and reduced diffusion behaviour of incorporated proteins for tethering densities higher than 10 mol% [26].

Polymer supported lipid bilayer membranes have been used in a large number of studies, for example, to investigate the incorporation of bovine rhodopsin [27], or to study the interactions between membrane-bound t-SNARE proteins and vesicle-bound v-SNARE proteins mediating cellular vesicle fusion [28]. By using fluorescence interference contrast measurements on a polymer-supported bilayer, it was shown that the rod-shaped t-SNARE/v-SNARE complex is embedded into the lipid bilayer in an upright orientation [29]. Polymer supported bilayers were also used to successfully incorporate a range of proteins in a functional form, for example, Adenylate Cyclase [30]. They have also been used to monitor the binding between Human Platelet Integrin and ligand-containing vesicles with high efficiency, demonstrating that little to no loss of functionality occurred once the protein was incorporated into a polymer-supported membrane [31].

### Effect of the Supporting Polymer on Bilayer Properties

Polymer supported and polymer tethered bilayers have been assembled on a wide variety of substrate materials, including glass, gold, and indium-tin oxide, and all have shown an effect on the final bilayer properties. Typically, a hydrophilic polymer of variable thickness and composition is used to enable the attachment of the lipid layer through either chemi- or physisorbtion [10].

Glass is one of the most popular substrates as its hydrophilic surface supports the formation of a highly hydrated sub-membrane reservoir. Various polymers have been used to form a hydrogel-cushion to support a lipid bilayer.

For example, methoxysilane-functionalised glass with a 80–90 Å thick maleic acid-based copolymer has been used [32]. A lipid bilayer composed of DMPC (dimyristoylphosphatidylcholin) and *N*-succinimidomyristidic ester was assembled onto this support via Langmuir-Blodgett (LB) followed by Langmuir-Schaefer (LS) transfer. *N*-succinimidomyristidic ester acts as a linker that integrates into the lipid bilayer due to its long aliphatic chain, but also covalently binds to the polymeric support, effectively tethering the bilayer to the polymer support. Both the addition of a polymer cushion to support the bilayer and tethering of the bilayer reduced the diffusion of phospholipids in the bilayer compared to diffusion rates observed in a solid supported bilayer without polymer cushion. A pure DMPC bilayer on an un-functionalised glass support showed lipid diffusion values around 4 μm^2^/s. Functionalisation of the glass with hydrogel reduced the diffusion to 3 μm^2^/s and the inclusion of 20 mol% anchoring agent reduced the diffusion coefficient to 1.3 μm^2^/s.

In a similar study, bilayers were deposited on glass substrates functionalised with a benzophenone silane [23]. The proximal bilayer leaflet was completed by LB transfer of a lipopolymer/phospholipid mixture, and the lipopolymer did covalently bind to the silane support via photo-crosslinking. The distal leaflet comprised various mixtures of DMPC and DMPE and was completed by LS transfer. The lipid bilayer was designed such that it was not reliant on electrostatic interactions, making it stable over a wider pH range. Studies on varying tethering polymer densities from 5% to 30% showed that an increase in the tethering density significantly reduced lateral lipid mobility at 40 °C in the outer leaflet from 18 μm^2^/s at 5% tether density to 1 μm^2^/s at 30% tethering density. At the same time, high tethering densities led to a decrease in the mobile fraction of lipids.

The tethering density also strongly affected lateral lipid diffusion in a lipopolymer supported SOPC (1-Stearoyl-2-oleoyl-*sn*-glycero-3-phosphocholine) bilayer [33]. A pure SOPC bilayer on a clean glass substrate was reported to have a diffusion coefficient of around 1.4 μm^2^/s, which was only marginally reduced for tethering densities up to 20 mol%. However, tethering densities of 50 mol% reduced the diffusion significantly to 0.4 μm^2^/s. The influence of the length of the lipopolymer was not thoroughly explored, but changing tether length while maintaining tethering density appeared to have no impact on lipid mobility. However, the homogeneous incorporation of integrin α_IIb_β_3_ was only possible in membranes supported by longer lipopolymer structures, whereas shorter lipopolymers caused the formation of distinct protein patches in the membrane.

Instead of tethering the lipid bilayer to a support via the incorporation of lipopolymers, a bilayer can also be attached to a polymer cushion through electrostatic means. For example, maleic acid-based copolymers with adjustable cushion thicknesses of up to 60 nm have been achieved by incorporation of ethene, propene, or octadecene side chains into the polymer [34]. Bilayers were formed from a range of naturally occurring phospholipids such as egg phosphatidylcholine. Increasing the thickness of the polymer cushion from 4 nm to 60 nm increased the diffusion coefficient from 0.26 to 1.1 μm^2^/s. The specific activity of incorporated proteins also increased with increasing cushion thickness.

Bilayers made from vesicles comprising a mixture of POPC (1-palmitoyl-2-oleoyl-*sn*-glycero-3-phosphocholine) and DOTAP (1,2-dioleoyl-3-trimethylammonium-propane) and assembled on 12 nm thick poly(amino acid methacrylate) cushions showed an even higher lipid mobility [35]. The lipids had no anchoring moieties and were held onto the polymer by electrostatic interactions between the cationic phospholipid DOTAP and the polymer cushion only. Pure POPC vesicles were unable to adsorb to the negatively charged polymer cushion and bilayers could only be formed upon the addition of 25 mol% DOTAP. The resulting bilayer had a mobile lipid fraction of 70% and lipid diffusion constants of up to 1.5 μm^2^/s [35].

Polyethyleneglycol (PEG) has been widely used as support for lipid bilayers. PEG-supported lipid bilayers can form aqueous reservoirs as thick as 10 nm underneath the membrane [36] and are well-suited to protein incorporation, especially if the protein has large trans-and sub-membrane domains. Hydration levels as high as 90% have been reported, and the supported membranes showed lipid diffusivity levels of up to 2.1 μm^2^/s and high tolerance for deformation, thereby suggesting a very rugged bilayer system [37]. In order to create a large sub-membrane space optimised for protein incorporation, a PEG-supported lipid bilayer system has been used, with the PEG layer grafted to the support and the lipid bilayer deposited by spin coating [37]. The resulting bilayer had a highly hydrated reservoir between the bilayer and the support with a thickness of around 55 Å and a lipid diffusion coefficient of around 2 μm^2^/s, only reduced by about 12% compared to a free floating lipid bilayer on the same support.

In other approaches, PEG chains have been grafted to a silicate substrate via silane terminal groups, with the lipid bilayer formed by LB transfer and vesicle fusion [24]. The resulting membrane was used to incorporate Cytochrome C, and analysis of the protein mobility showed three categories of diffusion speed (fast, slow, and immobile), with 80% of the proteins maintaining some degree of mobility regardless of tether density.

Hydrogels provide an alternative support material. Highly crosslinked layers of p(PFPA-*co*-MABP) on indium tin oxide with a dry thickness of about 60 nm and a mean roughness of 0.4 nm have been used to support lipid bilayers [38]. The mesh size of the hydrogel was designed such that it was smaller than the cytochrome c oxidase incorporated into the membrane, preventing the protein from migrating out of the lipid bilayer into the membrane support underneath. However, fluorescence experiments showed that the protein appeared to be immobilised in the membrane.

In another example, a protein-functionalised bilayer was assembled by functionalising a surface with a polyacrylamide-based hydrogel modified with nitrilotriacetic acid, which could act as a binding motif for cytochrome c oxidase. After binding of the protein, a lipid bilayer was assembled around it [38]. The resulting membrane had good sealing properties of 1–5 MΩ·cm^2^, and the functionality of the enzyme was shown electrochemically by the addition of cytochrome c. However, these good electrical sealing properties are rare for polymer supported membranes. Typically, they have shown lower values, which impedes the investigation of ion transport processes using these architectures.

## 3. Tethered Bilayer Lipid Membranes

**Figure 3:**
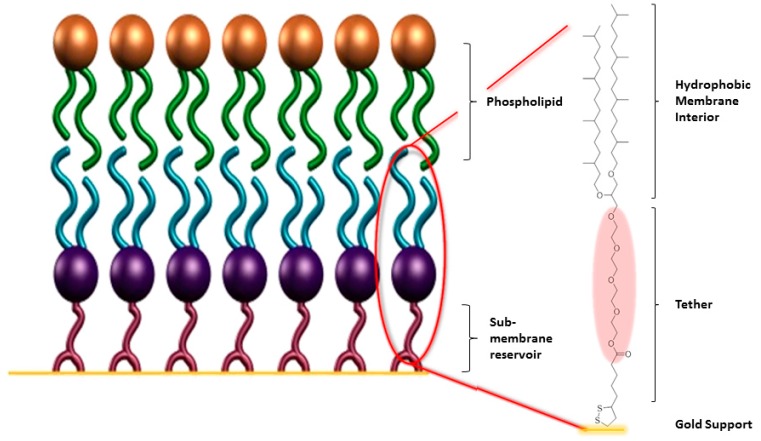
Schematic of a gold-supported tBLM with a fully tethered proximal leaflet (blue/purple) and a distal leaflet assembled from phospholipid (green/bronze). The frequently used anchorlipid DPhyTL is shown [39], which consists of a lipoic acid anchor, a teraethylene-glycol spacer, and a phytanyl lipid group.

tBLMs are an alternative approach to create a solid supported membrane including a reservoir between support and bilayer. In contrast to polymer supported membranes, tBLMs typically show high electrical resistance values and a higher long-term stability. A tBLM is a solid supported lipid bilayer [10], where the inner leaflet is separated from the support through a spacer unit, which also anchors the membrane covalently to the surface (Figure 3). In most cases, thiol anchors for the attachment to gold surfaces have been used [39,40,41], but there have also been examples for tBLMs on silicon surfaces using silane chemistry, phosphoric acid chemistry for aluminium surfaces, and thiol chemistry on mercury as a surface [42,43,44]. Gold is the most commonly used substrate, since it can easily be functionalised by thiol- and sulfur-based anchor moieties, and can also be used as an electrode in surface analytical methods. The outer membrane leaflet is typically formed by rapid solvent exchange [45], or fusion of the monolayer with lipid vesicles [46].

Ideally, a tBLM should mitigate the disadvantages of a polymer supported bilayer such as the difficulty of assembly, requiring polymer formation followed by LB/LS transfer, and the high defect density. They should at the same time maintain bilayer fluidity and provide a sufficiently well-hydrated sub-membrane domain to accommodate incorporated proteins.

tBLMs have been used in a large number of studies, ranging from the analysis of membrane structure and function, or the study of membrane-protein interactions to the investigation of membrane proteins embedded inside the membrane. Different tBLMs vary mainly in their tethering density and in the chemical structure of the tethering lipid used. These factors significantly influence the structural and functional parameters of the resulting bilayer. The composition of the outer leaflet can be adjusted as desired, thus allowing the creation of tailored biomimetic interfaces.

The composition of a lipid membrane is of significant importance in biological processes. For example, the accumulation of β-lactogobulin, a small globular protein, at a lipid interface was found to strongly depend on the phospholipids present in the membrane, as well as on the structure of the protein (denatured/native). Additionally, cholesterol in the membrane enabled protein penetration into the bilayer [47]. A mercury supported tethered model membrane system has been developed with the intention of being used to study the effect of antimicrobial peptides on the lipid bilayer structure and function [44]. A wide range of studies investigating membrane pores, pore forming peptides, and ion channels has been carried out using tBLMs such as the incorporation of ligand-gated ion channels [48], alpha-hemolysin [49,50], valinomycin [43,51], and gramicidin [42,52]. A tethered bilayer platform was also used to study the structure of the HIV-1 Gag protein as well as its interaction with lipid bilayers [53].

Amyloid β-oligomers and other misfolded oligomers and proteins are known to be a significant factor in the development of Parkinson’s [54] and Alzheimer’s disease [55]. tBLMs have been used to investigate the effect of these peptides on the cellular membrane, showing that oligomer accumulation at the membrane interface caused an increase in ion transport across the membrane [56]. As neural networks strongly rely on electrochemical gradients to function, this has shed light on the development of neurodegenerative diseases. Other studies have shown tBLMs to be a valid model to incorporate functional proteins such as ubiquinol oxidase into the lipid bilayer [57].

tBLMs have also been demonstrated to be viable platforms for highly adaptable biosensors by functionalising incorporated ion channels [41], and have also been used in studies to determine potential impacts of novel substances such as nanoparticles on the cellular membrane [58]. In another biosensing experiment, embedding a ferrocene-valinomycin redox sensor in a tBLM enabled the detection of ascorbic acid and sodium thiosulfate [59].

Tethered membranes have also been assembled on nanostructured substrates designed for surface enhanced infrared absorption spectroscopy (SEIRAS) [60]. Despite the increased surface roughness required for SEIRAS, a bilayer can be formed which is sufficiently sealing to allow ion channel studies. The use of SEIRAS enabled the elucidation of structural details of the incorporated protein that would otherwise not be seen. This is of significant importance as it provides an alternative to crystallographic techniques which required dried protein crystals where the proteins are no longer in their native environment and may therefore not be in their natural conformation.

### 3.1. Effect of the Tether Structure on Bilayer Properties

The chemical nature of the sub-membrane space has a significant impact on both the structure of the lipid bilayer as well as the functional incorporation of membrane components. Physical parameters such as bilayer impedance [40], bilayer fluidity [45], and hydration of the sub-membrane space [61,62] have been used to characterise different tBLM architectures. Ideally, and in order to mimic a natural membrane, a tBLM should have a high electrical impedance, a low capacitance, as well as high fluidity and high sub-membrane hydration. The electrical properties ensure that ion transport across the membrane is mainly due to the function of embedded protein or peptides, while the hydration is essential to allow for protein function. However, increases in membrane hydration and fluidity are generally accompanied by a reduction of the electrical sealing properties, resulting from a higher defect density [61,63]. The sub-membrane space of a tBLM is dominated by the structure of the tether-segment of the anchorlipid and the anchoring group itself, which is responsible for grafting the lipid onto the solid support.

The anchoring group can be adapted depending on the substrate. Surfaces that have been explored include gold [61,62,64], mercury [51,65,66], aluminium oxide [43], and silicon dioxide [42] as well as glass, indium tin oxide, and quartz [46]. As an alternative to using a single molecule to act as both a lower lipid leaflet and tethering agent, biotin-avidin interactions have been used to tether a bilayer to the surface [67]. Similarly, S-layer proteins [68] and DNA tethering have been explored as a method to construct more stable model membrane systems [69,70,71], but will not be discussed here. The structures of the various different tether-lipid structures that have been used on gold surfaces are compared in Figure 4.

While the anchoring group is usually only changed to fit the desired substrate, the spacer segment separating the lipid from the solid support can be varied to influence properties such as hydration and membrane fluidity. There is some evidence to suggest that using a thiol or disulphide anchor affects the packing density and resulting bilayer properties [48], but most research in this area focuses on changing the tether segment rather than the anchoring group to achieve increased sub-membrane hydration.

There has been a wide range of studies investigating the structural changes of the sub-membrane space as a consequence of changing the chemical composition and length of the tether segments. Examples have been summarised in Table 1. Commonly used techniques to study the membrane structure include neutron scattering [61], surface plasmon resonance, atomic force microscopy [74], and polarization modulation infrared reflection absorption spectroscopy (PM-IRRAS) [75]. Especially neutron scattering can give high-resolution structural information of the lipid bilayer system in an aqueous environment. For example, information about membrane thickness, composition, and hydration can be gained. Functional studies are often conducted using electrochemical impedance spectroscopy [61].

Neutron scattering studies have shown that the commonly used tethered lipid DPhyTL [39] has a poorly hydrated spacer segment [61], containing only 5 Volume-% water underneath the membrane, despite the fact that polyethylene glycol is known to be water soluble.

The low level of hydration in DPhyTL-based tBLMs has also been investigated using PM-IRRAS, a surface sensitive technique applicable to thin films [75]. The measurements showed that the tether segments of DPhyTL take on a coiled conformation, exposing the hydrophobic carbon segments of the tether to the surrounding medium. This hydrophobic conformation along with the high packing density of the self-assembled monolayer (SAM) causes low hydration of the sub-membrane region. At the same time, this architecture leads to highly insulating membranes and by reducing the size of the electrode surface, and thereby limiting the number of defect sites, the impedance of fully tethered tBLMs based on DPhyTL can achieve impedance values as high as 5.5 GΩ [78].

Sub-membrane hydration can be changed by modifying the spacer unit, and longer spacers typically lead to higher hydration levels [61]. The use of a thiol anchoring group instead of a disulphide and a slight increase in tether length from four to six ethylene glycol units increased membrane hydration from 5% in DPhyTL to around 20% in DPhyHT (Figure 4) and allowed a significantly higher rate of ion transport through a membrane without reduction in bilayer impedance [48,61].

Increasing the number of ethylene glycol units in the tether segment to eight resulted in an increase of sub-membrane hydration from 5% to 40% [61]. However, the increasing tether length also led to a significantly larger number of membrane defects, indicated by the increase in water incorporation into the distal leaflet and alkyl chains of the proximal leaflet and reduced electrical sealing properties.

Increasing tether length from four to eight ethylene glycol units on a mercury-supported tBLM significantly increased ion storage capacity under the membrane [66]. While mercury-supported tBLMs do not allow the use of surface-sensitive techniques, they do provide significantly higher membrane fluidity, avoiding some of the issues associated with tBLMs [65,66]. The use of mercury instead of gold as a supporting element allowed better functional incorporation of membrane active peptides such as gramicidin and the large antimicrobial polymer alamethicin [66].

However, increased tether length resulted in increased leakage of charged species across the membrane. This means that while a mercury-supported tBLM can be used for the incorporation of membrane proteins, this system is not well-suited for biosensing applications. The increased propensity to defect formation with increasing tether length also confirmed molecular dynamics simulations suggesting that longer tethers will form more disordered bilayers [63].

A fully tethered proximal leaflet enables the formation of highly electrically sealing bilayers, and this type of bilayer is most suitable for the analysis of channel and pore-forming proteins with no significant sub-membrane domains such as gramicidin or alpha-hemolysin. These systems are also suited to the study of ion transporters such as valinomycin where non-specific transport across the membrane must be as low as possible and the membrane fluidity as well as the mobility of incorporated components are not of significant interest. A fully tethered proximal leaflet also serves as an excellent platform for amperometric biosensors due to the low background current leakage resulting from the high electrical resistance of these bilayers [59].

However, a fully tethered proximal leaflet on a solid support is not well suited for protein incorporation as there is little space underneath the membrane for sub-membrane domains of proteins, thus possibly hindering incorporation and function. Furthermore, due to the packing density of the proximal leaflet, possible structural rearrangements of proteins are limited. In addition to spatial constraints imposed by a densely tethered proximal leaflet, the sub-membrane domain is typically poorly hydrated which can also negatively affect protein incorporation and function. The fluidity of the bilayer is also adversely impacted by the high tether density, hindering diffusion of proteins incorporated into the membrane and not allowing for significant structural rearrangements of the membrane.

To allow for a more flexible lipid bilayer and increase sub-membrane hydration, a bulkier anchoring group, sometimes referred to as a self-diluting tether-lipid, may be used instead of lipoic acid or a thiol, effectively reducing the packing density of the SAM. This approach resulted in significantly increased sub-membrane hydration around 60% using the DPhyHDL lipid (Figure 4), with much less reduction in bilayer resistance compared to longer tether molecules [61]. In addition to the significant increase in sub-membrane hydration, incorporated proteins are also able to transport ions across the membrane at significantly higher rates [50].

### 3.2. Sparsely Tethered Bilayer Lipid Membranes

In addition to lengthening the tether segment or changing the anchor group, it is also possible to achieve lower tethering density by diluting the SAM via the incorporation of small molecules into the proximal leaflet, thus competing with the anchorlipid for space on the gold support [62]. This creates a layer with a lower tethering density, offering more flexibility and space within. Most commonly, small molecules such as β-mercaptoethanol (βME) have been used in combination with an anchorlipid [62]. In addition to providing a more spacious water containing sub-membrane domain, sparsely tethered lipid bilayers with unsaturated phospholipids (DOPC, 1,2-dioleoyl-*sn*-glycero-3-phosphocholine) completing the bilayer have been shown to be more fluid than fully tethered membranes, with diffusion coefficients up to 7 μm^2^·s^−1^ in the distal leaflet, approaching values similar to natural cell membranes [45].

Hydration levels of up to 75 vol% hydration have been achieved by using sparsely tethered membranes, and these levels depend on the type of lipid used to complete the outer leaflet and tethering density of the lower leaflet [62]. However, high levels of hydration typically can lead to a significant reduction in impedance, likely due to higher propensity for defect formation. For example, 64% hydration of the sub-membrane space has been achieved with a tethering density of 30% WC14 and the outer leaflet completed with DPPC (1,2-dipalmitoyl-*sn*-glycero-3-phosphocholine). While a fully tethered layer had a resistance of 440 kΩ·cm^2^, dilution of the tether-lipid reduced the membrane resistance to 93 kΩ·cm^2^ [62]. Lengthening the tether by two ethylene glycol units (FC16 in Figure 4) did not adversely affect bilayer sealing qualities or membrane hydration at 30% tethering density (diluted with βME), but also caused no significant increase in sub-membrane hydration [76]. The increase in tether length did, however, enable better bilayer formation when using charged phospholipids to complete the bilayer, thereby allowing lipid packing densities similar to those in cellular membranes.

As alternatives to βME, thiol teminated tetraethylene glycol molecules have been used as backfiller combined with a TEG-DP anchorlipid (Figure 4), resulting in membrane resistances above 2 MΩ·cm^2^ for anchorlipid densities as low as 20% [73]. The need for a dilute sub-membrane space and a consequential increase in sub-membrane hydration has also been demonstrated using a cholesterol-tethered membrane with mercaptohexanol acting as a diluting agent [77]. Such lipid bilayers with 80% tethering density achieved membrane resistances around 11 MΩ·cm^2^, and ion transport across the membrane due to incorporated valinomycin and gramicidin was only possible in diluted tethering architectures. Sparsely tethered membranes with a proximal leaflet diluted by mercaptohexanol remained stable for over 60 h, making them suitable for longer term studies and biosensing applications [77].

In addition to providing a more suitable sub-membrane space for protein incorporation, sparsely tethered membranes also increase bilayer fluidity, especially in the distal leaflet. Diffusion in the proximal leaflet is hindered by the presence of the anchorlipids. For example, diffusion coefficients for the outer leaflet of 7 μm^2^·s^−1^ have been measured, with diffusion in the proximal leaflet reduced to 2 μm^2^·s^−1^ [45]. Generally, diffusivities are reported as average values of the proximal and distal leaflet diffusivities due to the difficulty of distinguishing between the two leaflets [45].

A tether-lipid molecule containing unsaturated alkyl chains (HC18) enabled higher diffusion rates of 4 μm^2^·s^−1^ than lipids such as DPhyTL and WC14/FC16 containing saturated or highly branched alkyl chains with diffusion coefficients of 2 μm^2^·s^−1^ [72]. The increased fluidity of the membrane is most likely due to the more disordered bilayer structure caused by the less flexible unsaturated alkyl chains. However, the tether segment in dilute HC18-based bilayers is able to coil up or tilt, resulting in a thinner sub-membrane reservoir of around 10 Å in HC18 compared to 15–18 Å in WC14 and FC16 [72]. This same trend can be observed with regards to the composition of the distal lipid. More ordered bilayer structures formed by DPhyPC show lower diffusion rates and higher membrane resistance than bilayers formed by POPC, for example [45].

HC18 at 70% tethering density led to average lipid diffusion rates of up to 4 μm^2^/s while also achieving 24% sub-membrane hydration at 30% tether density [72]. This further demonstrates the high flexibility with which membrane properties can be fine-tuned depending upon the desired application.

## 4. Conclusions

Tethered lipid bilayer membranes have been developed to the point that they can mitigate the flaws initially associated with these systems-poor sub-membrane hydration and low bilayer fluidity. Combined with the ease with which tBLMs can be assembled, they are ideal platforms for membrane protein studies as well as highly specific biosensors.

The composition of the tBLMs can be adjusted to form bilayers which support single-channel pore protein studies, but they can also be adjusted to have lipid diffusion rates similar to those in natural systems, which can then be used to reproduce membrane processes not related to charge transport. More complex membrane assemblies can also be prepared in which both membrane resistance and membrane fluidity are high enough to enable protein incorporation as well as transport studies.

## Figures and Tables

**Figure 4:** Chemical structure of various tether-lipids.

**Table:** 

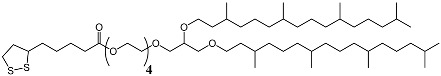	DPhyTL [39,47,50,64]
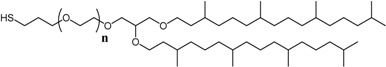	DPhyTT (*n* = 4) [61]
	DPhyHT (*n* = 6) [61]
	DPhyOT (*n* = 8) [61]
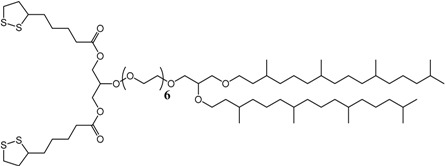	DPhyHDL [50,61]
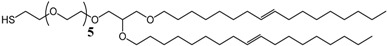	HC18 [72]
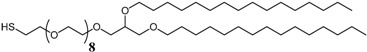	FC16 [72]
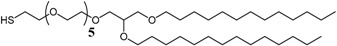	WC14 [49,56,62,72]
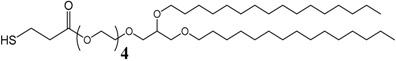	TEG-DP [73]

**Table 1:** Bilayer parameters of different tethered and sparsely tethered membrane systems.

Proximal Leaflet Composition	Distal Leaflet Composition	Sub-Membrane Hydration (Volume-%)	Membrane Resistance (MΩ·cm^2^)	Average Lipid Diffusion Constant (μm^2^·s^−1^)
DPhyTL [61]	DPhyPC	5	3–55	N/A
DPhyTT [61]	DPhyPC	13	3–15	N/A
DPhyHT [61]	DPhyPC	21	2–35	N/A
DPhyOT [61]	DPhyPC	40	0.01–0.09	N/A
DPhyHDL [61]	DPhyPC	65	0.2–6.5	N/A
30% WC14 [45]	DOPC/DPhyPC	N/A	N/A	4.1/3.1
40% WC14 [62]	DPPC	64 [39]	0.09	
70% WC14 [72]	DOPC	N/A	N/A	1.9
FC 16 [76]	DPhyPC	53	0.1	N/A
30% FC16	DOPC/DPhyPC	53 [76]	N/A	3.6/2.5 [45]
70% FC16 [72]	DOPC	N/A	N/A	1.6
30% HC18 [72]	DOPC	21	N/A	4.1
20% TEG-DP [73]	POPC	N/A	2	N/A
80% Cholesterol-tether [77]	*E. coli* lipid extract	N/A	11	N/A

## References

[1] Palczewski K., Kumasaka T., Hori T., Behnke C.A., Motoshima H., Fox B.A., Le Trong I., Teller D.C., Okada T., Stenkamp R.E. (2000). Crystal structure of rhodopsin: A G protein-coupled receptor. Science.

[2] Leutenegger M., Lasser T., Sinner E.K., Robelek R. (2008). Imaging of G protein-coupled receptors in solid-supported planar lipid membranes. Biointerphases.

[3] Nakaya M., Chikura S., Watari K., Mizuno N., Mochinaga K., Mangmool S., Koyanagi S., Ohdo S., Sato Y., Ide T. (2012). Induction of Cardiac Fibrosis by beta-Blocker in G Proteinin-dependent and G Protein-coupled Receptor Kinase 5/beta-Arrestin2-dependent Signaling Pathways. J. Biol. Chem..

[4] Kristiansen K. (2004). Molecular mechanisms of ligand binding, signaling, and regulation within the superfamily of G-protein-coupled receptors: Molecular modeling and mutagenesis approaches to receptor structure and function. Pharmacol. Ther..

[5] Cheng S.H., Gregory R.J., Marshall J., Paul S., Souza D.W., White G.A., Oriordan C.R., Smith A.E. (1990). Defective intracellular-transport and processing of CFTR is the molecular basis of most cystic-fibrosis. Cell.

[6] Emery A.E.H. (2002). The muscular dystrophies. Lancet.

[7] Klabunde T., Hessler G. (2002). Drug design strategies for targeting G-protein-coupled receptors. Chembiochem. Eur. J. Chem. Biol..

[8] Guo D., Hillger J.M., Ijzerman A.P., Heitman L.H. (2014). Drug-Target Residence Time—A Case for G Protein-Coupled Receptors. Med. Res. Rev..

[9] Knobloch J., Suhendro D.K., Zieleniecki J.L., Shapter J.G., Köper I. (2015). Membrane—Drug interactions studied using model membrane systems. Saudi J. Biol. Sci..

[10] Sackmann E. (1996). Supported membranes: Scientific and practical applications. Science.

[11] Tanaka M., Sackmann E. (2005). Polymer-supported membranes as models of the cell surface. Nature.

[12] Winterhalter M. (2000). Black lipid membranes. Cur. Opin. Colloid Interface Sci..

[13] Chan Y.H.M., Boxer S.G. (2007). Model membrane systems and their applications. Cur. Opin. Chem. Biol..

[14] Castellana E.T., Cremer P.S. (2006). Solid supported lipid bilayers: From biophysical studies to sensor design. Surface Sci. Rep..

[15] Johnson S.J., Bayerl T.M., McDermott D.C., Adam G.W., Rennie A.R., Thomas R.K., Sackmann E. (1991). Structure of an adsorbed dimyristoylphosphatidylcholine bilayer measured with specular reflection of neutrons. Biophys. J..

[16] Koenig B.W., Krueger S., Orts W.J., Majkrzak C.F., Berk N.F., Silverton J.V., Gawrisch K. (1996). Neutron Reflectivity and Atomic Force Microscopy Studies of a Lipid Bilayer in Water Adsorbed to the Surface of a Silicon Single Crystal. Langmuir.

[17] Rinia H.A., Snel M.M.E., van der Eerden J., de Kruijff B. (2001). Visualizing detergent resistant domains in model membranes with atomic force microscopy. FEBS Lett..

[18] Kiessling V., Crane J.M., Tamm L.K. (2006). Transbilayer effects of raft-like lipid domains in asymmetric planar bilayers measured by single molecule tracking. Biophys. J..

[19] Tokumasu F., Jin A.J., Feigenson G.W., Dvorak J.A. (2003). Nanoscopic lipid domain dynamics revealed by atomic force microscopy. Biophys. J..

[20] Wildman K.A.H., Lee D.K., Ramamoorthy A. (2003). Mechanism of lipid bilayer disruption by the human antimicrobial peptide, LL-37. Biochemistry.

[21] Urbina J.A., Pekerar S., Le H.B., Patterson J., Montez B., Oldfield E. (1995). Molecular order and dynamics of phosphatidycholine bilayer-membranes in the presence of cholesterol, ergosterol and lanosterol—A comparative study using H-2-NMR, C-13-NMR and P-31-NMR Spectroscopy. Biochim. Et Biophys. Acta-Biomembr..

[22] Rinia H.A., Kik R.A., Demel R.A., Snel M.M.E., Killian J.A., van der Eerden J., de Kruijff B. (2000). Visualization of highly ordered striated domains induced by transmembrane peptides in supported phosphatidylcholine bilayers. Biochemistry.

[23] Naumann C.A., Prucker O., Lehmann T., Rühe J., Knoll W., Frank C.W. (2002). The polymer-supported phospholipid bilayer: Tethering as a new approach to substrate-membrane stabilization. Biomacromolecules.

[24] Wagner M.L., Tamm L.K. (2000). Tethered polymer-supported planar lipid bilayers for reconstitution of integral membrane proteins: Silane-polyethyleneglycol-lipid as a cushion and covalent linker. Biophys. J..

[25] Su Z., Jiang Y., Velázquez-Manzanares M., Leitch J.J., Kycia A., Lipkowski J. (2013). Electrochemical and PM-IRRAS studies of floating lipid bilayers assembled at the Au (111) electrode pre-modified with a hydrophilic monolayer. J. Electroanal. Chem..

[26] Deverall M.A., Gindl E., Sinner E.K., Besir H., Ruehe J., Saxton M.J., Naumann C.A. (2005). Membrane Lateral Mobility Obstructed by Polymer-Tethered Lipids Studied at the Single Molecule Level. Biophys. J..

[27] Subramaniam V., Alves I.D., Salgado G.F.J., Lau P.-W., Wysocki R.J., Salamon Z., Tollin G., Hruby V.J., Brown M.F., Saavedra S.S. (2005). Rhodopsin Reconstituted into a Planar-Supported Lipid Bilayer Retains Photoactivity after Cross-Linking Polymerization of Lipid Monomers. J. Am. Chem. Soc..

[28] Wagner M.L., Tamm L.K. (2001). Reconstituted Syntaxin1A/SNAP25 Interacts with Negatively Charged Lipids as Measured by Lateral Diffusion in Planar Supported Bilayers. Biophys. J..

[29] Kiessling V., Tamm L.K. (2003). Measuring Distances in Supported Bilayers by Fluorescence Interference-Contrast Microscopy: Polymer Supports and SNARE Proteins. Biophys. J..

[30] Rossi C., Chopineau J. (2007). Biomimetic tethered lipid membranes designed for membrane-protein interaction studies. Eur. Biophys. J..

[31] Purrucker O., Gonnenwein S., Fortig A., Jordan R., Rusp M., Barmann M., Moroder L., Sackmann E., Tanaka M. (2007). Polymer-tethered membranes as quantitative models for the study of integrin-mediated cell adhesion. Soft Matter.

[32] Beyer D., Elender G., Knoll W., Kühner M., Maus S., Ringsdorf H., Sackmann E. (1996). Influence of anchor lipids on the homogeneity and mobility of lipid bilayers on thin polymer films. Angew. Chem. Int. Ed. Engl..

[33] Purrucker O., Förtig A., Jordan R., Tanaka M. (2004). Supported Membranes with Well-Defined Polymer Tethers—Incorporation of Cell Receptors. ChemPhysChem.

[34] Renner L., Pompe T., Lemaitre R., Drechsel D., Werner C. (2010). Controlled enhancement of transmembrane enzyme activity in polymer cushioned supported bilayer membranes. Soft Matter.

[35] Blakeston A.C., Alswieleh A.M., Heath G.R., Roth J.S., Bao P., Cheng N., Armes S.P., Leggett G.J., Bushby R.J., Evans S.D. (2015). New Poly(amino acid methacrylate) Brush Supports the Formation of Well-Defined Lipid Membranes. Langmuir.

[36] Ye Q., Konradi R., Textor M., Reimhult E. (2009). Liposomes Tethered to Omega-Functional PEG Brushes and Induced Formation of PEG Brush Supported Planar Lipid Bilayers. Langmuir.

[37] Hertrich S., Stetter F., Rühm A., Hugel T., Nickel B. (2014). Highly Hydrated Deformable Polyethylene Glycol-Tethered Lipid Bilayers. Langmuir.

[38] Kibrom A., Roskamp R.F., Jonas U., Menges B., Knoll W., Paulsen H., Naumann R.L. (2011). Hydrogel-supported protein-tethered bilayer lipid membranes: A new approach toward polymer-supported lipid membranes. Soft Matter.

[39] Schiller S.M., Naumann R., Lovejoy K., Kunz H., Knoll W. (2003). Archaea analogue thiolipids for tethered bilayer lipid membranes on ultrasmooth gold surfaces. Angew. Chem. Int. Ed..

[40] Köper I. (2007). Insulating tethered bilayer lipid membranes to study membrane proteins. Mol. Biosyst..

[41] Cornell B.A., BraachMaksvytis V.L.B., King L.G., Osman P.D.J., Raguse B., Wieczorek L., Pace R.J. (1997). A biosensor that uses ion-channel switches. Nature.

[42] Atanasov V., Knorr N., Duran R.S., Ingebrandt S., Offenhäusser A., Knoll W., Köper I. (2005). Membrane on a chip: A functional tethered lipid bilayer membrane on silicon oxide surfaces. Biophys. J..

[43] Roskamp R.F., Vockenroth I.K., Eisenmenger N., Braunagel J., Koeper I. (2008). Functional tethered bilayer lipid membranes on aluminum oxide. ChemPhysChem.

[44] Becucci L., Guidelli R. (2014). Mercury-supported biomimetic membranes for the investigation of antimicrobial peptides. Pharmaceuticals.

[45] Shenoy S., Moldovan R., Fitzpatrick J., Vanderah D.J., Deserno M., Losche M. (2010). In-plane homogeneity and lipid dynamics in tethered bilayer lipid membranes (tBLMs). Soft Matter.

[46] Knoll W., Köper I., Naumann R., Sinner E.-K. (2008). Tethered bimolecular lipid membranes—A novel model membrane platform. Electrochim. Acta.

[47] Junghans A., Champagne C., Cayot P., Loupiac C., Köper I. (2011). Probing Protein—Membrane Interactions Using Solid Supported Membranes. Langmuir.

[48] Vockenroth I.K., Atanasova P.P., Long J.R., Jenkins A.T.A., Knoll W., Köper I. (2007). Functional incorporation of the pore forming segment of AChR M2 into tethered bilayer lipid membranes. Biochim. Biophys. Acta Biomembr..

[49] McGillivray D.J., Valincius G., Heinrich F., Robertson J.W., Vanderah D.J., Febo-Ayala W., Ignatjev I., Lösche M., Kasianowicz J.J. (2009). Structure of functional Staphylococcus aureus α-hemolysin channels in tethered bilayer lipid membranes. Biophys. J..

[50] Vockenroth I.K., Atanasova P.P., Jenkins A.T.A., Koper I. (2008). Incorporation of alpha-hemolysin in different tethered bilayer lipid membrane architectures. Langmuir.

[51] Becucci L., Moncelli M.R., Naumann R., Guidelli R. (2005). Potassium ion transport by valinomycin across a Hg-supported lipid bilayer. J. Am. Chem. Soc..

[52] Jadhav S.R., Sui D., Garavito R.M., Worden R.M. (2008). Fabrication of highly insulating tethered bilayer lipid membrane using yeast cell membrane fractions for measuring ion channel activity. J. Colloid Interface Sci..

[53] Datta S.A., Heinrich F., Raghunandan S., Krueger S., Curtis J.E., Rein A., Nanda H. (2011). HIV-1 Gag extension: Conformational changes require simultaneous interaction with membrane and nucleic acid. J. Mol. Biol..

[54] Haass C., Selkoe D.J. (2007). Soluble protein oligomers in neurodegeneration: Lessons from the Alzheimer’s amyloid beta-peptide. Nat. Rev. Mol. Cell Biol..

[55] Lesné S., Koh M.T., Kotilinek L., Kayed R., Glabe C.G., Yang A., Gallagher M., Ashe K.H. (2006). A specific amyloid-[beta] protein assembly in the brain impairs memory. Nature.

[56] Valincius G., Heinrich F., Budvytyte R., Vanderah D.J., McGillivray D.J., Sokolov Y., Hall J.E., Losche M. (2008). Soluble Amyloid beta-Oligomers Affect Dielectric Membrane Properties by Bilayer Insertion and Domain Formation: Implications for Cell Toxicity. Biophys. J..

[57] Jeuken L.J.C., Connell S.D., Henderson P.J.F., Gennis R.B., Evans S.D., Bushby R.J. (2006). Redox Enzymes in Tethered Membranes. J. Am. Chem. Soc..

[58] Goreham R.V., Thompson V.C., Samura Y., Gibson C.T., Shapter J.G., Köper I. (2015). Interaction of silver nanoparticles with tethered bilayer lipid membranes. Langmuir.

[59] Braunagel J., Junghans A., Köper I. (2011). Membrane-based sensing approaches. Austr. J. Chem..

[60] Kozuch J., Steinem C., Hildebrandt P., Millo D. (2012). Combined Electrochemistry and Surface-Enhanced Infrared Absorption Spectroscopy of Gramicidin A Incorporated into Tethered Bilayer Lipid Membranes. Angew. Chem. Int. Ed..

[61] Junghans A., Köper I. (2010). Structural Analysis of Tethered Bilayer Lipid Membranes. Langmuir.

[62] McGillivray D.J., Valincius G., Vanderah D.J., Febo-Ayala W., Woodward J.T., Heinrich F., Kasianowicz J.J., Losche M. (2007). Molecular-scale structural and functional characterization of sparsely tethered bilayer lipid membranes. Biointerphases.

[63] Liu C., Faller R. (2012). Conformational, Dynamical. and Tensional Study of Tethered Bilayer Lipid Membranes in Coarse-Grained Molecular Simulations. Langmuir.

[64] Atanasov V., Atanasova P.P., Vockenroth I.K., Knorr N., Koper I. (2006). A molecular toolkit for highly insulating tethered bilayer lipid membranes on various substrates. Bioconj. Chem..

[65] Moncelli M., Becucci L., Schiller S.M. (2004). Tethered bilayer lipid membranes self-assembled on mercury electrodes. Bioelectrochemistry.

[66] Becucci L., Faragher R.J., Schwan A. (2015). The effect of the hydrophilic spacer length on the functionality of a mercury-supported tethered bilayer lipid membrane. Bioelectrochemistry.

[67] Taylor J.D., Linman M.J., Wilkop T., Cheng Q. (2009). Regenerable Tethered Bilayer Lipid Membrane Arrays for Multiplexed Label-Free Analysis of Lipid—Protein Interactions on Poly (dimethylsiloxane) Microchips Using SPR Imaging. Anal. Chem..

[68] Schrems A., Kibrom A., Kupcu S., Kiene E., Sleytr U., Schuster B. (2011). Bilayer lipid membrane formation on a chemically modified S-layer lattice. Langmuir.

[69] Chung M., Lowe R.D., Chan Y.-H.M., Ganesan P.V., Boxer S.G. (2009). DNA-tethered membranes formed by giant vesicle rupture. J. Struct. Biol..

[70] Chung M., Boxer S.G. (2011). Stability of DNA-Tethered Lipid Membranes with Mobile Tethers. Langmuir.

[71] Hughes L.D., Boxer S.G. (2013). DNA-Based Patterning of Tethered Membrane Patches. Langmuir.

[72] Budvytyte R., Valincius G., Niaura G., Voiciuk V., Mickevicius M., Chapman H., Goh H.Z., Shekhar P., Heinrich F., Shenoy S. (2013). Structure and Properties of Tethered Bilayer Lipid Membranes with Unsaturated Anchor Molecules. Langmuir.

[73] Basit H., Van der Heyden A., Gondran C., Nysten B., Dumy P., Labbe P. (2011). Tethered Bilayer Lipid Membranes on Mixed Self-Assembled Monolayers of a Novel Anchoring Thiol: Impact of the Anchoring Thiol Density on Bilayer Formation. Langmuir.

[74] Vockenroth I.K., Rossi C., Shah M.R., Köper I. (2009). Formation of tethered bilayer lipid membranes probed by various surface sensitive techniques. Biointerphases.

[75] Leitch J., Kunze J., Goddard J.D., Schwan A.L., Faragher R.J., Naumann R., Knoll W., Dutcher J.R., Lipkowski J. (2009). *In Situ* PM-IRRAS Studies of an Archaea Analogue Thiolipid Assembled on a Au(111) Electrode Surface. Langmuir.

[76] Heinrich F., Ng T., Vanderah D.J., Shekhar P., Mihailescu M., Nanda H., Lösche M. (2009). A New Lipid Anchor for Sparsely Tethered Bilayer Lipid Membranes. Langmuir.

[77] Kendall J.K., Johnson B.R., Symonds P.H., Imperato G., Bushby R.J., Gwyer J.D., van Berkel C., Evans S.D., Jeuken L.J. (2010). Effect of the Structure of Cholesterol-Based Tethered Bilayer Lipid Membranes on Ionophore Activity. ChemPhysChem.

[78] Vockenroth I.K., Fine D., Dodobalapur A., Jenkins A.T.A., Köper I. (2008). Tethered bilayer lipid membranes with giga-ohm resistances. Electrochem. Commun..

